# Elamipretide Improves Mitochondrial Function in Mitochondrial Trifunctional Protein‐Deficient Mice and Human Fibroblasts

**DOI:** 10.1002/jimd.70132

**Published:** 2026-01-07

**Authors:** Eduardo Vieira Neto, Meicheng Wang, Austin J. Szuminsky, Lethicia Ferraro, Shakuntala Basu, Xuejun Zhao, Anuradha Karunanidhi, Yudong Wang, Jerry Vockley

**Affiliations:** ^1^ Genetic and Genomic Medicine Division, Department of Pediatrics, UPMC Children's Hospital of Pittsburgh University of Pittsburgh Pittsburgh Pennsylvania USA; ^2^ Department of Pediatrics Children's Neuroscience Institute, School of Medicine, University of Pittsburgh Pittsburgh Pennsylvania USA; ^3^ Department of Biological Sciences, Kenneth P. Dietrich School of Arts and Sciences University of Pittsburgh Pittsburgh Pennsylvania USA; ^4^ School of Medicine, Federal University of Rio Grande do Sul Porto Alegre Rio Grande do Sul Brazil; ^5^ Department of Human Genetics, School of Public Health, Center for Rare Disease Therapy, UPMC Children's Hospital of Pittsburgh University of Pittsburgh Pittsburgh Pennsylvania USA

**Keywords:** cardiolipin, elamipretide, electron transport chain complex proteins, fatty acid oxidation, mitochondria, mitochondrial trifunctional protein deficiency, multiprotein energy complex, oxidative phosphorylation

## Abstract

Mitochondrial trifunctional protein (TFP) deficiency is an inherited disorder of long‐chain fatty acid β‐oxidation (FAO). TFP is a heteromeric enzyme composed of two α and two β‐subunits. Despite early detection and dietary treatment, TFP deficiency patients often develop hypoglycemia, rhabdomyolysis, cardiomyopathy, and peripheral neuropathy. Degenerative retinopathy and milder peripheral neuropathy occur in patients with an isolated deficiency of the αTFP subunit of long‐chain 3‐hydroxyacyl‐CoA dehydrogenase (LCHAD) activity. Triheptanoin treatment improves most complications, but not peripheral neuropathy and retinopathy. Notably, TFP also carries a fourth enzymatic function involved in cardiolipin remodeling, which we previously found to be impaired in TFP/LCHAD deficiency. We therefore tested whether elamipretide, a synthetic cardiolipin‐binding peptide, could improve mitochondrial function and cardiolipin levels in βTFP‐deficient mice and patient‐derived fibroblasts. Mice were treated with elamipretide delivered by osmotic minipump and challenged with treadmill exercise or cold stress after fasting. βTFP‐deficient mice treated with elamipretide showed improved exercise endurance, but cold tolerance was not altered. Liver mitochondria from male βTFP‐deficient mice demonstrated improved FAO‐ETC enzyme activities. However, cardiolipin content and composition were unchanged. In patient fibroblasts, elamipretide produced a possible genotype‐dependent increase in mitochondrial bioenergetics and a reduction in ROS. These results support a mechanism in which elamipretide stabilizes between FAO enzymes and ETC complexes, thereby improving mitochondrial function independently of changes in cardiolipin levels. Elamipretide thus emerges as a potential therapeutic agent for TFP/LCHAD deficiency, warranting further preclinical studies.

## 1 Introduction

Mitochondrial fatty acid β‐oxidation (FAO) is the biochemical pathway by which fatty acids released from stored fats generate energy through production of tricarboxylic acid cycle (TCA) intermediates and reducing equivalents for oxidative phosphorylation (OXPHOS) [1]. FAO is the primary energy source during periods of limited glucose availability, such as fasting, fever, and exercise, but even when glucose is available, it is the primary source of energy for the heart, skeletal muscles, and kidneys [2]. In reality, FAO is essential for energy production by most organs, except the brain and erythrocytes. Additionally, FAO is crucial for the liver to produce ketone bodies, which are utilized by nearly all tissues, including the brain [3]. Fatty acid oxidation disorders (FAODs) are the most prevalent group of conditions identified by newborn screening, with an incidence in several European and US studies ranging from 6.0 to 16.4 per 100 000 live‐born infants [4].

Catabolism of long‐chain fatty acids involves import into mitochondria via the carnitine cycle, followed by removal of successive two‐carbon units (acetyl‐CoA) via four enzymatic steps. The first intramitochondrial step is catalyzed by very‐long‐chain acyl‐CoA dehydrogenase (VLCAD, EC 1.3.8.9), while mitochondrial trifunctional protein (TFP) performs the next three reactions. TFP is a heteromeric enzyme composed of two α‐subunits and two β‐subunits, encoded by the *HADHA* and *HADHB* genes, respectively [5]. The HADHA protein contains the 2‐enoyl‐CoA hydratase (ECH, EC 4.2.1.74) and the long‐chain 3‐hydroxyacyl‐CoA dehydrogenase (LCHAD, EC 1.1.1.211) activities, while the HADHB protein contains the 3‐ketoacyl‐CoA thiolase activity (KAT, EC 2.3.1.16). Monolysocardiolipin acyltransferase‐1 (MLCLAT‐1), a fourth enzymatic activity recently mapped to the HADHA subunit, catalyzes transfer of long‐chain acyl‐CoAs, preferentially linoleoyl‐CoA, to lyso‐, de‐acylated, cardiolipin (CL), generating tetra‐acylated unsaturated mature CL, a process called remodeling [6, 7]. CL is a unique phospholipid that makes up to 15%–20% of the inner mitochondrial membrane (IMM) phospholipids [8]. It is the only known dimeric phospholipid, and its unusual structure is essential for maintaining mitochondrial cristae morphology and the organization and stabilization of membrane‐associated proteins, especially when it comes to the formation of mitochondrial electron transport chain (ETC) supercomplexes, large complexes of metabolic proteins that engage in substrate shuttling to increase enzymatic activity and optimize OXPHOS [9, 10, 11, 12]. Furthermore, TFP interacts with ETC supercomplexes to enhance the functional efficiency of OXPHOS [13]. The involvement of TFP in these functions suggests that OXPHOS dysfunction could play a role in the pathogenesis of generalized *HADHA* TFP deficiency (OMIM #609015), generalized *HADHB* TFP deficiency (OMIM #620300), and isolated LCHAD deficiency (OMIM #609016), especially the development of retinopathy, peripheral neuropathy, and cardiomyopathy.

Elamipretide is an aromatic‐cationic synthetic tetrapeptide that interacts with CL [14]. The drug has been shown to reduce mitochondrial reactive oxygen species (ROS) production and to increase mitochondrial respiration, membrane potential, and ATP production in several animal model studies [15, 16, 17]. Clinical trials to assess its potential therapeutic benefits have been performed in several conditions, including Barth syndrome [18], primary mitochondrial myopathy [19], heart failure with reduced ejection fraction [20, 21], and stent revascularization in patients with atherosclerotic renal artery stenosis [22]. Recently, elamipretide received accelerated FDA approval for the treatment of Barth syndrome in adult and pediatric patients weighing at least 30 kg (~66 lb), based on data from the TAZPOWER trial, which showed improvements in knee extensor muscle strength [18]. We have previously reported a decrease in CL levels in mitochondrial isolates of fibroblasts from TFP/LCHAD‐deficient patients and in the liver from a βTFP‐mutant mouse model [23]. These results have led us to consider elamipretide as a therapeutic option for TFP/LCHAD deficiency.

Our objective in this study was to examine the ability of elamipretide to improve in vitro and in vivo FAO, ETC enzyme activities, and CL levels in TFP/LCHAD deficiency.

## 2 Materials and Methods

### 2.1 Mice

All animal studies were conducted in the vivarium of Rangos Research Center at the UPMC Children's Hospital of Pittsburgh (Pittsburgh, PA, USA) in accordance with protocols approved by the University of Pittsburgh Institutional Animal Care and Use Committee (IACUC). A βTFP‐mutant mouse model was previously created using *N*‐ethyl‐*N*‐nitrosourea mutagenesis [24]. These mice, C57BL/6J‐*Hadhb* ^m1Ytc^, have a missense mutation in exon 14 (c.1210T>A;p.M404K) of *HADHB*. Their phenotype includes reduced lifespan, cold sensitivity, hepatic steatosis after prolonged fasting, and cardiac fibrosis and arrhythmia. They were bred by crossing homozygous males with heterozygous females, and housed in group cages in an SPF, temperature, and light (12‐h light–dark cycle) controlled facility. We previously demonstrated reduced CL levels in this model, supporting its relevance for testing CL‐targeted therapeutics [23]. For most experiments, 6‐ to 7‐month‐old mice were studied, as this age corresponds to middle adulthood and permits evaluation of elamipretide when the functional decline due to TFP deficiency is fully established.

### 2.2 Elamipretide Treatment

To evaluate tolerability and efficacy, we tested multiple dosing regimens. Elamipretide acetate salt was purchased from BOC Sciences (Shirley, NY, USA) and dissolved in phosphate‐buffered saline (PBS). The dosing strategy was based on allometric scaling from the clinical TAZPOWER trial dose of 40 mg/day (~8.2 mg/kg/day in mice, calculated per Nair and Jacob [25]). In practice, an initial pilot with intraperitoneal (IP) injections of 100 μg/g/day proved acutely toxic, resulting in rapid mortality of three female C57BL/6J‐*Hadhb* ^m1Ytc^ mice within minutes of the first injection. Based on this observation, the dose was reduced to 25 μg/g/day, which was well tolerated. At this dose, six mice (three males and three females) were treated by daily IP injection for 8 days, while four female controls received PBS. A longer‐term trial using daily subcutaneous (SC) injections at 25 μg/g/day for 8 weeks resulted in high mortality (66.7%). Based on these findings, subsequent studies employed continuous SC delivery of elamipretide using ALZET micro‐osmotic pumps (model 1004; DURECT, Cupertino, CA, USA), which provided a calculated 3 μg/g/day for 28 days in a 25 g mouse. Control mice received pumps filled with PBS. The pump regimen was well tolerated and was therefore used for all subsequent efficacy studies. For these studies, mice were separated by sex, with approximately three to four animals per group receiving either elamipretide or PBS.

### 2.3 Cold Sensitivity

Cold sensitivity was evaluated at the end of drug treatment—8 days for IP injection and 28 days for micro‐osmotic pump‐delivery as previously described [26, 27]. Mice were then fasted for 18 h, followed by exposure to cold at 9°C. Rectal temperature was measured before exposure and then hourly. If rectal temperature dropped below 25°C or a period of 4 h was reached (whichever occurred first), mice were sacrificed with isoflurane, and the liver was collected for further biochemical analyses.

### 2.4 Treadmill Exercise

Only mice treated with elamipretide delivered by micro‐osmotic pump and controls (PBS) were submitted to treadmill exercise. Mice were tested after 25–26 days of elamipretide treatment (2–3 days before the cold sensitivity test). A starting speed of 9 m/min on the treadmill led to rapid exhaustion in female mice (both treated and control); subsequently, the starting speed was set at 5 m/min for 5 min, then increased to 7 m/min until exhaustion. Wild‐type (WT) mice were assessed using a more strenuous protocol appropriate for their normal physiological capacity. After an initial warm‐up at 8 m/min, speed was increased in stages up to 14 m/min, followed by small incremental accelerations until exhaustion. The total distance run and time to exhaustion were recorded for all animals.

### 2.5 Human Fibroblasts

Human fibroblast lines were previously established from skin biopsy samples of TFP/LCHAD‐deficient patients (FB822, FB830, FB847, and FB861) for other clinical testing (Tables S1 and S2). Controls were dermal fibroblast lines from unaffected individuals: FB826 from a 40‐year‐old healthy woman and FB902 from a 22‐year‐old healthy male (Table S3).

Fibroblasts were grown as previously described at 37°C and 5% CO_2_ in high‐glucose‐ and l‐glutamine‐containing DMEM supplemented with 10% fetal calf serum, 100 units/mL penicillin, and 100 μg/mL streptomycin (complete DMEM) [28, 29]. The passage number of cells used in this study was between 4 and 7.

### 2.6 Mitochondria Isolation

Mitochondria were isolated from mouse liver and hindlimb skeletal muscles, homogenized at 4°C in separation buffer (50 mM phosphate buffer, pH 8.0, 2.5% glycerol, 250 mM sucrose, and 1 mM EDTA) supplemented with freshly added complete EDTA‐free protease inhibitor cocktail (Roche Diagnostics, Mannheim, Germany) as previously described [30], with minor modifications. Nuclei and cell debris were first removed by a 10‐min centrifugation at 700 × *g*, and then the supernatant was collected and centrifuged at 14 000 × *g* for 15 min at 4°C. Mitochondrial fractions were maintained at −80°C until analysis.

### 2.7 Blue‐Native Polyacrylamide Gel Electrophoresis (BN‐PAGE) and 2D‐Electrophoresis

BN‐PAGE of mouse liver and hindlimb skeletal muscle isolated mitochondria was done as previously described [31]. Briefly, mitochondria were permeabilized with digitonin followed by the addition of Coomassie Blue G250 (Bio‐Rad) in ε‐aminocaproic acid solution and centrifugation at 14 000 × *g* for 30 min at 4°C. The supernatant was directly loaded onto a 3%–12% gradient native PAGE Bis‐Tris, 1.0 mm, precast mini‐gel (Invitrogen, Carlsbad, CA, USA), subjected to electrophoresis, followed by Coomassie Blue G250 staining to visualize oxidative phosphorylation (OXPHOS) complexes and supercomplexes. For normalization, each lane was loaded with 60 μg of protein. For second dimension separation, a strip of the gel corresponding to a single sample well was rotated 90° and placed on a 4%–15% gradient Criterion precast SDS‐PAGE gel (Bio‐Rad, Hercules, CA, USA) and subjected to electrophoresis. Following electrophoresis, western blotting was performed as previously described [23, 32]. Primary antibodies used were mouse monoclonal IgG_1_ anti‐HADHA (Proteintech, Rosemont, IL, USA) and mouse monoclonal IgG_2a_ anti‐HADHB (Santa Cruz Biotech, Santa Cruz, CA, USA). Protein banding was visualized using Pierce ECL Western Blotting Substrate Kit (ThermoFisher Scientific, Rockford, IL, USA). Protein band density was quantified from BN‐PAGE gels and Western blots by densitometry using Fiji (ImageJ) [33].

#### 2.7.1 In Situ Gel Staining for ETC Complex I Activity

Strips corresponding to the sample wells were excised from BN‐PAGE gels and incubated with a reaction buffer system specific for ETC complex I. The activity staining procedure was done as described [13, 31, 34].

#### 2.7.2 In Situ Gel Staining for Complex V (ATPase) Activity

After BN‐PAGE, complex V (ATPase) activity was located on the gel by incubation in a 34 mM Tris–HCl buffer, pH 8.6, containing 270 mM glycine, 14 mM MgSO_4_, 0.2% Pb(NO_3_)_2_, and 8 mM ATP, pH 7.8, as described [34].

### 2.8 Mitochondrial ETC Activity Assays

#### 2.8.1 ETC Complex Activity Assays in Mouse Liver Isolated Mitochondria

Enzyme activities of individual mitochondrial respiratory chain complexes were determined spectrophotometrically using established methods, with minor adaptations as noted below.

- Complex I + III activity (NADH‐cytochrome *c* reductase): Activity was assayed in 50 mM phosphate buffer (pH 7.4) containing 50 μM NADH, 1 μM KCN (complex IV inhibitor), 50 μM oxidized coenzyme Q1 (CoQ1), and 30 μM cytochrome *c*. The reduction of cytochrome *c* was monitored as an increase in absorbance at 550 nm [35].
- Complex II (succinate‐DCPIP reductase): The assay was performed in 20 mM Tris–HCl buffer (pH 7.5) with 200 μM succinate as electron donor and 50 μM 2,6‐dichlorophenolindophenol (DCPIP) as the electron acceptor. The reaction mixture also contained 100 mM KCl, 37.5 μM rotenone (complex I inhibitor), 10 μM KCN, and 10–20 μM oxidized CoQ1. Activity was measured as the decrease in absorbance at 605 nm due to DCPIP reduction, and initial reaction rates were calculated [36].
- Complex IV (cytochrome *c* oxidase): Activity was measured in 20 mM phosphate buffer (pH 7.4) containing 6 μM reduced cytochrome *c* and 100 mM KCl. The decrease in absorbance at 550 nm was recorded, as previously described [31, 37].
- Complex V: activity was assayed as ATPase by quantifying inorganic phosphate released from ATP hydrolysis using the molybdenum blue method, as described by Katewa and Katyare [38]. Briefly, assays were carried out in 1 mL 20 mM Tris–HCl buffer, pH 7.9, containing 3 mM MgCl_2_(6H_2_O), 50 mM KCl, and 3 mM ATP. Reaction was initiated with the addition of 1.5–3.0 μg of mitochondria extract protein and incubated for 15 min at 25°C. The reaction was stopped with 100 μL 75% trichloroacetic acid, centrifuged (2000 × *g*, 3 min), and inorganic phosphate (Pi) quantified in 0.3 mL of the supernatant.

Enzyme activities were monitored as changes in absorbance per minute at the indicated wavelengths (ΔAbs/min), with identical protein amounts and assay conditions applied across groups to enable valid relative comparisons.

#### 2.8.2 FAO‐ETC Bridging Assay

FAO‐ETC bridging activity was assayed as previously described [31]. Briefly, permeabilized mouse liver mitochondria were incubated with palmitoyl‐CoA as substrate in a reaction mixture containing oxidized CoQ1, cytochrome *c*, KCN, and electron transfer flavoprotein (ETF). Reduction of cytochrome *c*, reflecting transfer of reducing equivalents from palmitoyl‐CoA to the ETC, was monitored as an increase in absorbance at 550 nm.

### 2.9 ROS Production

#### 2.9.1 Isolated Mouse Liver Mitochondria

ROS generation from FAO to ETC was measured using the non‐fluorescent probe 2′,7′‐dichlorodihydrofluorescein diacetate (DCFH‐DA) [39, 40]. The assay was performed with 30 μg of isolated mitochondria added to 1 mL of buffer containing 20 mM Tris–HCl pH 7.4, 100 mM KCl, 5 mM CoQ1, 5 mM NAD, 3 mM ETF, 5 μM DCFH‐DA, and 2 mM MgCl_2_. The reaction was then initiated by adding 1 mM palmitoyl‐CoA or C20‐CoA. Measurement of the fluorescent intensity was performed with an excitation wavelength of 485 nm and an emission wavelength of 520 nm.

#### 2.9.2 Human Patient‐Derived Fibroblasts

The following patient‐derived fibroblasts were studied: FB822, FB861, and FB847 (Tables S1 and S2). Controls were dermal fibroblasts from an unaffected female (FB826; Table S3). TFP/LCHAD‐deficient and control cell lines were grown to 90%–100% confluency in T175 or T182 flasks and harvested by trypsinization. The harvested cells were seeded into new T175 or T182 flasks and cultivated for 72 h in the presence of 0, 12.5, and 25 nM of elamipretide. Cells were again harvested by trypsinization, and four to six aliquots containing each 3 × 10^5^ cells/200 μL were transferred to 2 mL microcentrifuge Eppendorf tubes. The contents of one vial (50 μg) of MitoSOX Red (Invitrogen) were dissolved in 13 μL DMSO (Invitrogen) to make 5 mM (5000 μM) stock solution. Stock solution was diluted in complete DMEM media to 15 μM, and 100 μL was transferred to each Eppendorf tube containing harvested cells to a final concentration of 5 μM. Cells were incubated for 20 min at 37°C, then 10 000 cells were analyzed in a FACSAria II flow cytometer (BD Biosciences, San Jose, CA, USA).

### 2.10 Mouse Liver Mitochondria Lipidomics

The method was as previously described [23]. Samples were extracted using Matyash extraction procedure [41]. The organic phase was dried and resuspended with 110 μL of a solution of 9:1 methanol:toluene containing deuterated internal standards (UltimateSPLASH ONE, Avanti Polar Lipids, Alabaster, AL, USA), 12‐[[(cyclohexylamino) carbonyl]amino]‐dodecanoic acid (CUDA), and supplemental standards. This was shaken (20 s), sonicated (5 min), centrifuged (2 min at 16 100 × *g*), and used for LC–MS/MS analysis. Samples were injected into a Vanquish UHPLC liquid chromatography system coupled to a Q‐Exactive HF orbital ion trap mass spectrometer (ThermoFisher Scientific) and analyzed in both positive and negative mode. An ACQUITY Premier BEH C18 column (1.7 μm, 2.1 × 50 mm) (Waters, Milford, MA, USA) was used to separate complex lipids. The separation was carried out with a flow rate of 0.8 mL/min and a column temperature of 65°C. For positive mode, the mobile phases consisted of: (A) 10 mM ammonium formate in 60:40 (v/v) acetonitrile:water + 0.1% formic acid, and (B) 10 mM ammonium formate in 90:10:0.1 (v/v/v) isopropanol:acetonitrile:water + 0.1% formic acid. For negative mode, the mobile phases consisted of: (A) 10 mM ammonium acetate in 60:40 (v/v) acetonitrile:water, and (B) 10 mM ammonium acetate in 90:10:0.1 (v/v/v) isopropanol:acetonitrile:water. The LC gradient was 0 min 15% (B), 0.75 min 30% (B), 0.98 min 48% (B), 4.00 min 82% (B), 4.13–4.50 min 99% (B), 4.58–5.50 min 15% (B). Data were collected with a scan range of 120–1700 *m/z* and 60 000 mass resolution.

### 2.11 Mitochondrial Bioenergetics

Cellular mitochondrial bioenergetics were measured as the oxygen consumption rate (OCR) in a Seahorse XFe96 Extracellular Flux Analyzer (Agilent Technologies, Santa Clara, CA, USA) as previously described [23]. Cells were seeded in uncoated 96‐well tissue culture microplates at a density of 30 000 cells/80 μL per well in complete DMEM. After attachment to the well surface for 24 h, they were treated for 72 h with elamipretide (0, 25, 50, or 100 nM; additional assays included 0, 10, 100, and 1000 nM) diluted in media with glucose. Prior to measurement, culture medium was replaced with Seahorse XF DMEM assay medium without glucose, supplemented with 1 mM pyruvate and 2 mM l‐glutamine, and cells were incubated for 1 h in a non‐CO_2_ incubator. Each fibroblast cell line was seeded in at least four biological replicates.

### 2.12 Statistics

Rectal temperatures of mice submitted to cold challenge were tested by simple linear regression, with P values obtained for slopes (rate of decline) and elevations (intercepts, baseline temperature). A simple mean imputation method was applied for animals that reached a rectal temperature ≤ 25°C [42]. Most direct comparisons across experimental groups were tested with an unpaired *t*‐test. Comparisons of mitochondrial bioenergetics in selected human fibroblasts were tested by two‐way ANOVA followed by Šídák's multiple comparisons test.

## 3 Results

### 3.1 Elamipretide Did Not Alter Cold Tolerance in βTFP Mutant Mice

**FIGURE 1:**
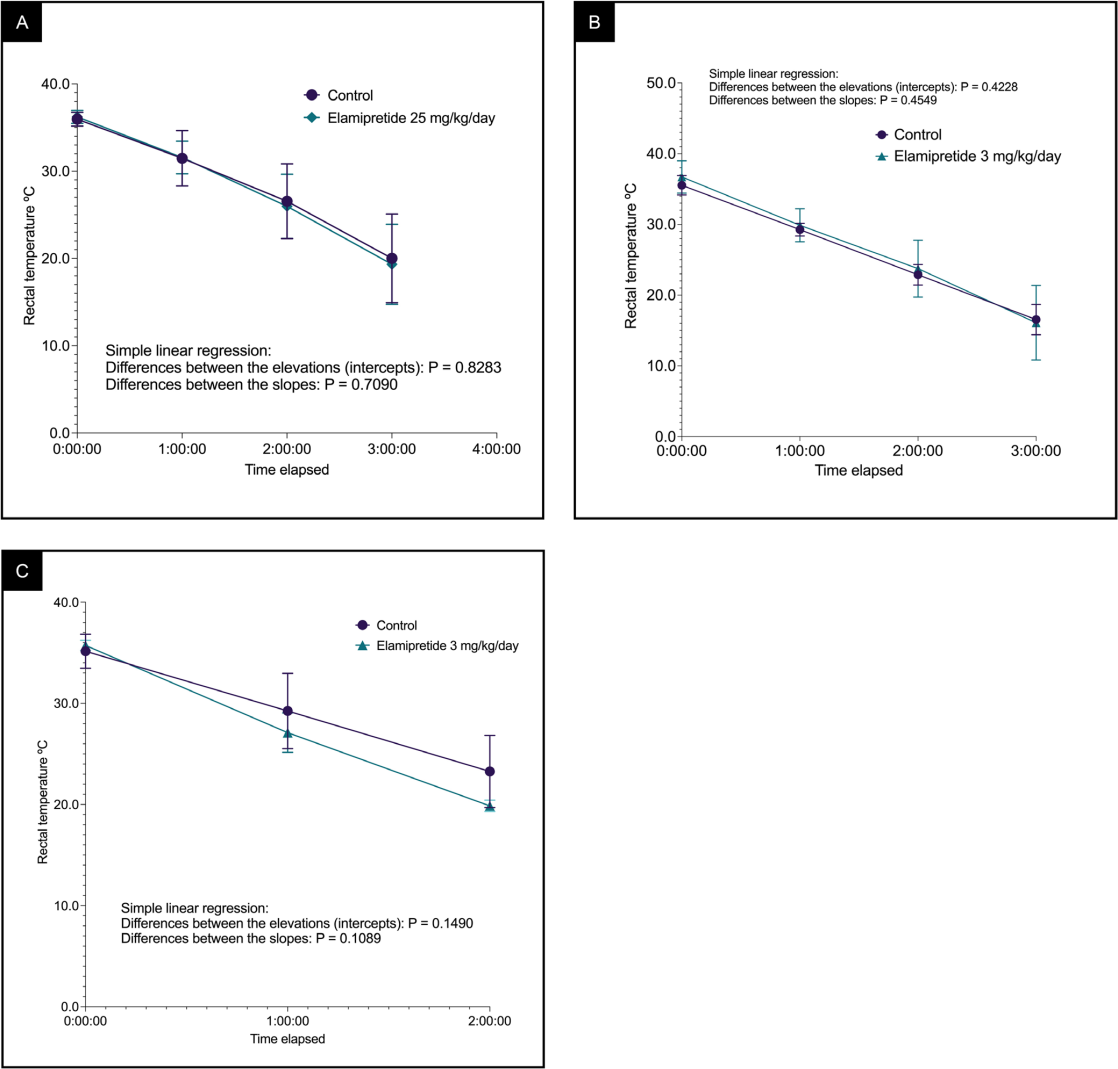
Rectal temperature curves of βTFP mutant mice treated with elamipretide or placebo (PBS). Mice were fasted for 18 h and then exposed to 9°C. (A) Daily IP injections of 25 μg/g/day elamipretide for 8 days, six treated and four control mice. Simple linear regression showed no significant differences in slopes (*p* = 0.7090) or intercepts (*p* = 0.8283). No inference regarding sex was made because all controls were female. (B) Male mice treated for 28 days by osmotic minipumps at ~3 μg/g/day, combined data from two independent experiments totaling eight treated and eight control mice. Intercepts (*p* = 0.4228) and slopes (*p* = 0.4549) were not significantly different, showing similar baseline temperatures and cooling rates between groups. (C) Female mice treated for 28 days by osmotic minipumps at ~3 μg/g/day, four treated and four control mice. Slopes (*p* = 0.1089) and intercepts (*p* = 0.1490) were not significantly different. For reference, C57BL/6J wild‐type mice tolerate 3–4 h cold stress without significant loss of body temperature [26, 27].

An exploratory trial showed that IP injection of elamipretide at 100 μg/g body weight was acutely lethal, with rapid mortality shortly after dosing. The dose was reduced to 25 μg/g/day for 8 days, tested primarily in female mice. This lower dose was well tolerated but showed no improvement in cold tolerance (Figure 1A). A longer‐term trial of daily SC injections at the same dose for 8 weeks resulted in high mortality (~67%) and was discontinued.

We therefore turned to continuous SC delivery via miniature implantable osmotic pumps for 28 days (~3 μg/g/day) in all subsequent experiments. Male and female mice were tested separately. In males, combined data from two independent experiments involving eight mice per group showed no significant difference between the treated and control groups (Figure 1B). Treated female mice also showed no improvement in cold tolerance (Figure 1C). Consistent with previous reports, WT C57BL/6J mice tolerate a 3–4 h cold stress without significant loss of body temperature [26, 27].

### 3.2 Elamipretide Treatment Improved Running Distance in βTFP‐Deficient Mice

**FIGURE 2:**
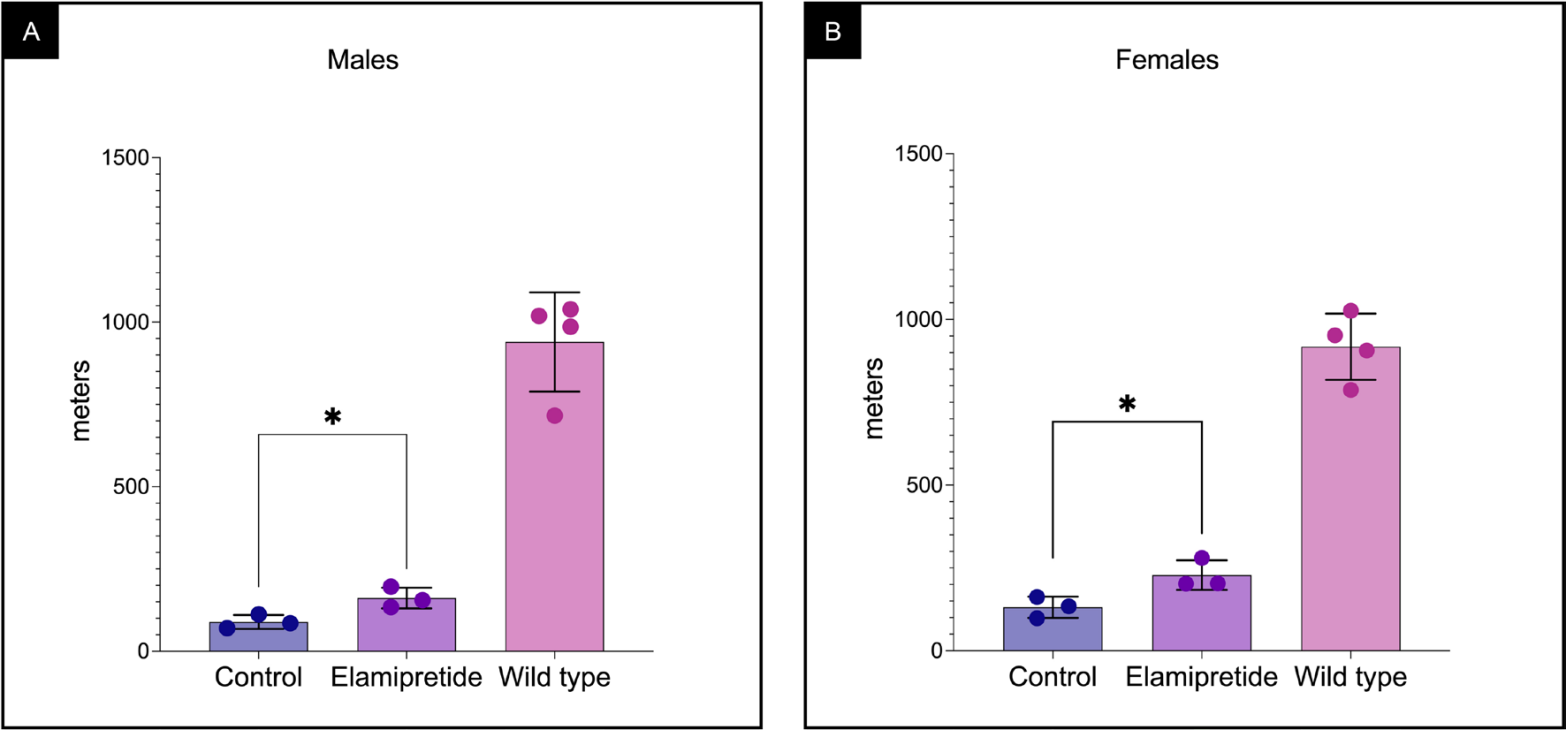
Distance run (meters) on a treadmill. βTFP mutant mice treated with elamipretide delivered by micro‐osmotic pumps and PBS‐treated mutant controls were subjected to treadmill exercise. Untreated male and female C57BL/6J wild‐type mice (4 per group) are shown for comparison. Among mutant mice, elamipretide‐treated animals (*n* = 3 per sex) ran significantly farther than untreated controls (*n* = 3 per sex). (A) Male mutant mice: *P* = 0.0297, unpaired *t*‐test. (B) Female mutant mice: *P* = 0.0380, unpaired *t*‐test.

Male βTFP‐deficient mice treated with elamipretide ran a mean distance of 162 m, a significantly longer distance than untreated males, mean of 89 m (Figure 2A). Treated female mutant mice ran 228 m, also significantly longer than untreated females, mean of 131 m (Figure 2B). For context, male C57BL/6J WT mice ran a mean ± SD of 940 ± 151 m, and female WT mice ran 918 ± 100 m under the more strenuous WT treadmill protocol.

### 3.3 Elamipretide Treatment Increased ETC Complexes and Supercomplexes in βTFP‐Deficient Mice

**FIGURE 3:**
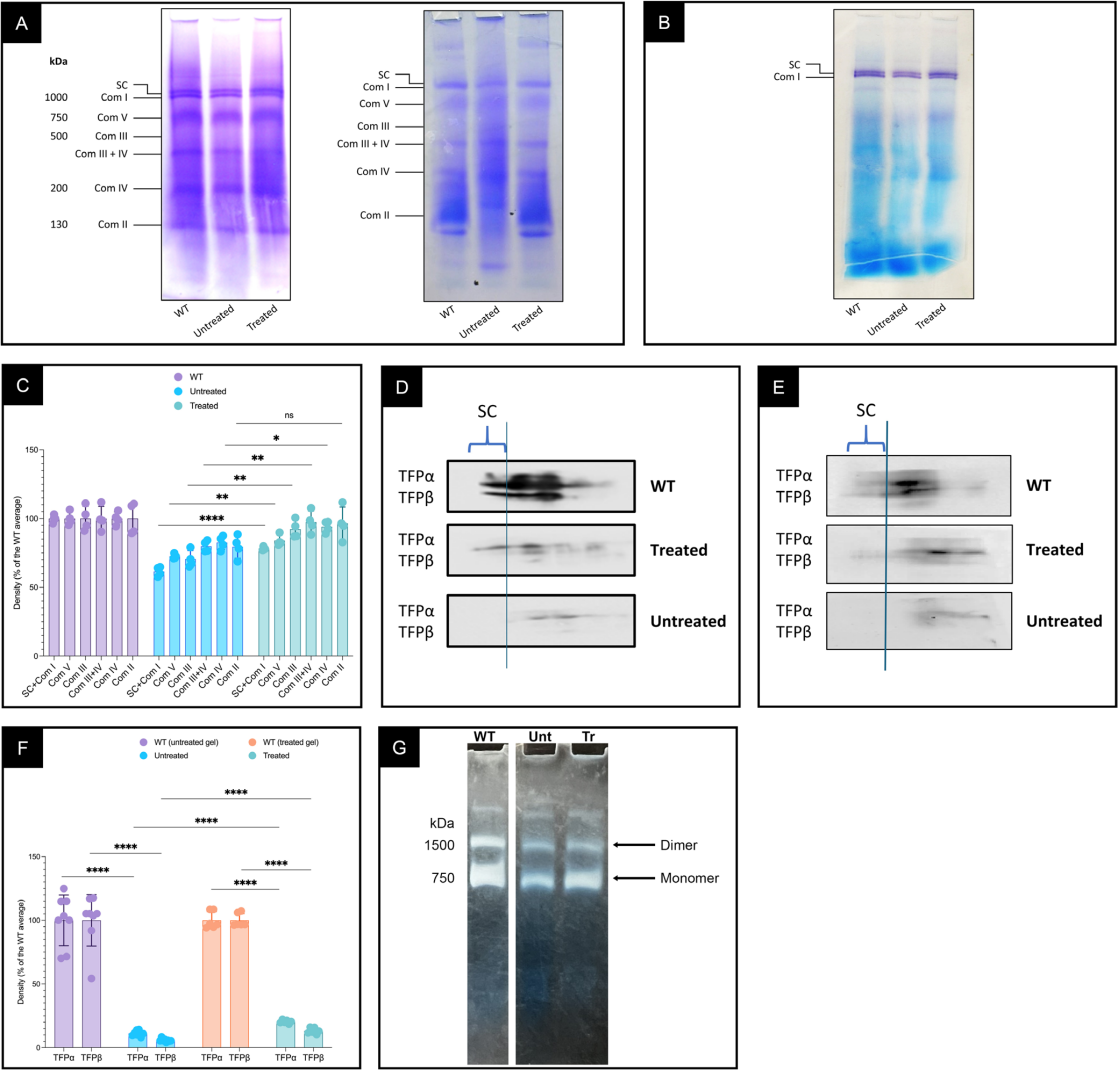
BN‐PAGE, Western blotting after 2D‐electrophoresis, and in‐gel staining for ETC complex activities in mitochondria from male βTFP mutant mice treated with elamipretide (osmotic minipumps) compared with untreated controls and C57BL/6J wild‐type (WT) mice. (A) BN‐PAGE of liver (left) and hindlimb skeletal muscle (right) mitochondria stained with Coomassie blue, showing increased ETC complexes and supercomplexes after treatment. (B) In‐gel complex I activity detected in complex I and in supercomplexes containing complex I, increased after treatment. (C) Quantification of protein band densities of complexes and supercomplexes in liver mitochondria extracts; four technical repeats were obtained per protein band, WT set to 100%, comparisons by unpaired *t*‐test. (D) and (E) 2D BN/SDS‐PAGE and Western blotting of liver (D) and hindlimb skeletal muscle (E) mitochondria probed with HADHA (TFPα) and HADHB (TFPβ) monoclonal antibodies. Untreated mutant mice showed reduced TFPα and TFPβ in the high‐molecular‐mass region, whereas the TFPα signal partially reappeared after treatment. (F) Quantification of colocalized TFPα and TFPβ in liver mitochondria increased from 12% and 6% to 20% and 13% of WT values after treatment, respectively (unpaired *t*‐test). (G) In‐gel complex V activity in liver mitochondria of βTFP mutant mice increased after treatment. All samples were run on the same gel. Lanes not pertinent to the current study were removed, and the remaining WT lane was repositioned adjacent to the βTFP mutant samples for clarity. A vertical blank interval indicates splice boundaries. The full, unprocessed gel is provided as Figure S3. *p* values: ns *p* > 0.05, *p ≤ 0.05, **p ≤ 0.01, ***p ≤ 0.001, and ****p ≤ 0.0001.

Mitochondrial ETC complexes and supercomplexes were reduced in βTFP‐deficient liver and hindlimb skeletal muscle mitochondria compared to WT, as demonstrated by BN‐PAGE (Figure 3A). Treatment of mutant animals with elamipretide increased ETC complexes and supercomplexes, as visualized by protein staining and in‐gel activity staining for all complexes except complex II (Figure 3B,C,G). 2D BN/SDS‐PAGE followed by western blotting showed reduced TFPα and TFPβ in high molecular mass complexes in βTFP‐deficient mitochondria (Figure 3D–F). Elamipretide treatment increased the amount of both subunits detected in the supercomplex region. Elamipretide also increased in‐gel complex V activity, with a more pronounced effect on the monomeric form than the dimer, the latter being generally considered the physiologic form (Figure 3G).

### 3.4 Elamipretide Treatment Increased Activities of ETC Complexes and FAO‐ETC Bridging in Mitochondria Extracts From βTFP‐Deficient Mice

**FIGURE 4:**
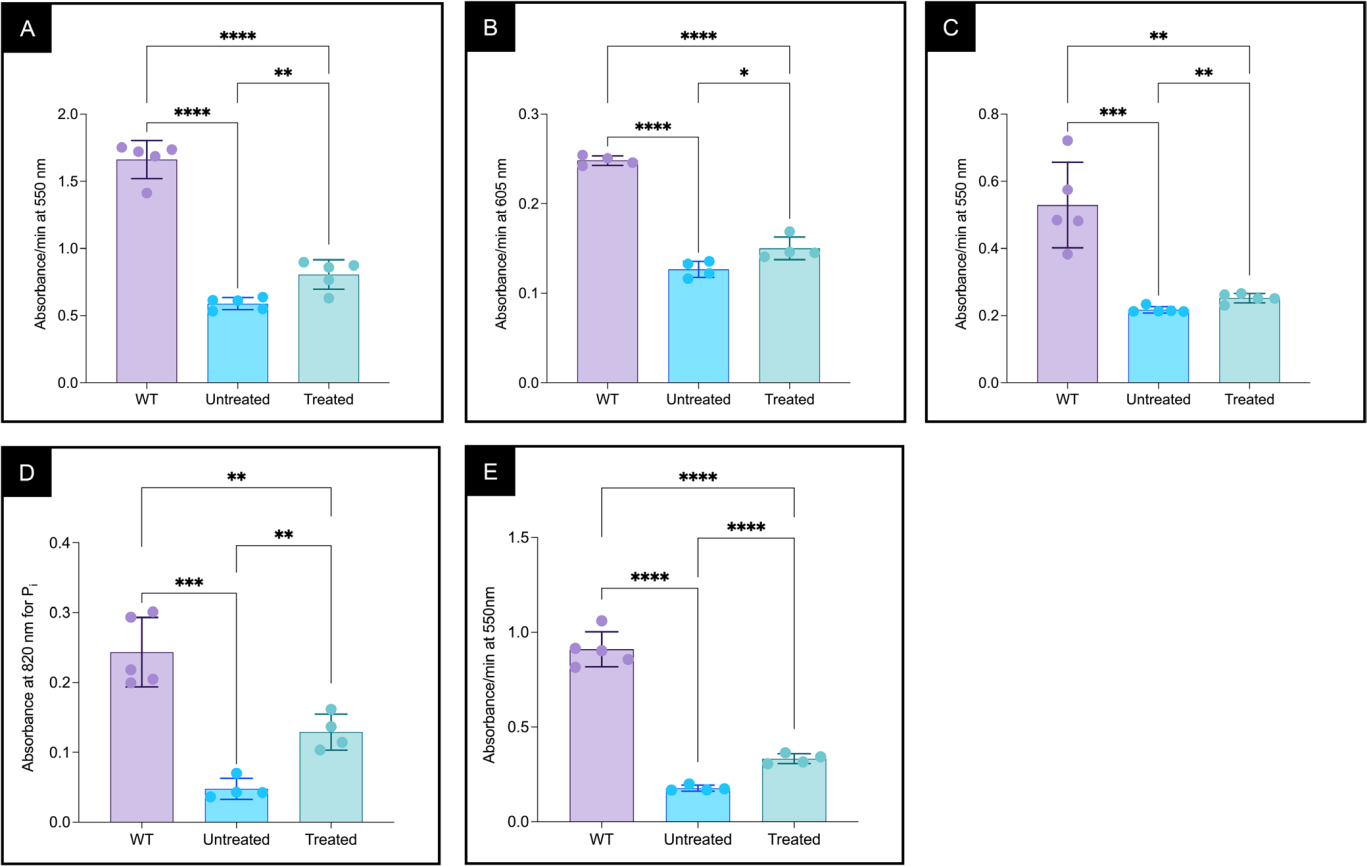
Complex I + III, II, IV, and V activities and FAO‐ETC bridging in liver mitochondria from male βTFP mutant mice treated with elamipretide (osmotic minipumps) compared with untreated mutants and C57BL/6J WT mice. (A) Complex I + III activity measured as cytochrome *c* reduction (increase in absorbance at 550 nm) in the presence of KCN (complex IV inhibitor) and CoQ1. (B) Complex II activity measured as DCPIP reduction (decrease in absorbance at 605 nm) using succinate as donor, in the presence of rotenone (complex I inhibitor), KCN, and CoQ1. (C) Complex IV activity measured as cytochrome *c* oxidation (decrease in absorbance at 550 nm). (D) Complex V activity measured as ATPase, monitored by inorganic phosphate (Pi) release using the molybdenum blue method (absorbance at 820 nm). (E) FAO‐ETC bridging assay with palmitoyl‐CoA as substrate. Elamipretide increased the activity of all ETC complexes tested and FAO‐ETC bridging. Comparisons were performed using an unpaired *t*‐test. *p* values: ns *p* > 0.05, *p ≤ 0.05, **p ≤ 0.01, ***p ≤ 0.001, and ****p ≤ 0.0001.

Liver mitochondria extracts from male βTFP‐deficient mice showed significantly decreased activities of complexes I + III, II, IV, and V compared to WT animals (Figure 4A–D). Treatment with elamipretide increased the activities of all four complexes (Figure 4A–D). In addition, the flux of reducing equivalents from palmitoyl‐CoA to complex III (FAO‐ETC bridging) was improved (Figure 4E), indicating enhanced function of both individual ETC complexes and the multiprotein energy complex (MPEC).

### 3.5 Elamipretide Treatment of TFP/LCHAD‐Deficient Patient‐Derived Fibroblasts Reduced ROS Production

**FIGURE 5:**
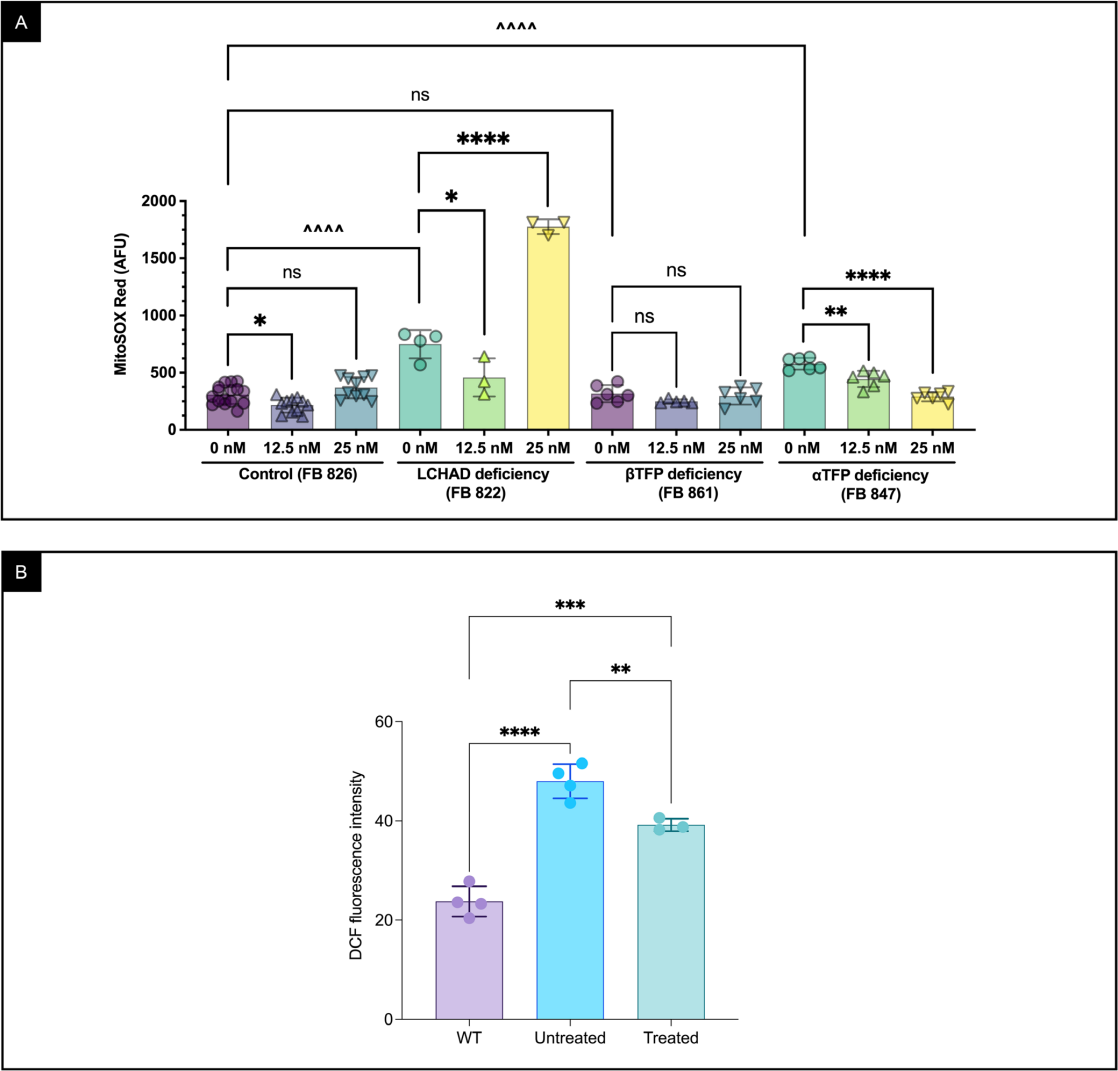
Mitochondrial ROS production in fibroblasts from TFP/LCHAD deficiency patients and control fibroblasts, and in liver mitochondria from βTFP mutant mice. (A) ROS levels in fibroblasts from patients with *HADHA* or *HADHB* variants compared to a control fibroblast line (FB826; healthy donor‐derived, ATCC PCS‐201‐012), measured as MitoSOX Red fluorescence by flow cytometry. Patient genotypes and clinical details are provided in Tables S1 and S2. (B) ROS levels in liver mitochondria extracts from male βTFP mutant mice treated with elamipretide delivered by osmotic minipumps, untreated βTFP mutants, and C57BL/6J WT mice. ROS was measured by the fluorochrome 2′,7′‐dichlorofluorescein (DCF). Comparisons were performed using an ordinary one‐way ANOVA with Tukey's multiple comparisons test for drug effects in fibroblasts, and an unpaired *t*‐test for untreated patient versus control fibroblasts, and liver mitochondria from βTFP mutant mice. Panel A: *p* values: ns *p* > 0.05; Tukey’s: *p ≤ 0.05, **p ≤ 0.01, ****p ≤ 0.0001; unpaired *t* test: ^^^^^^ *p* ≤ 0.0001. Panel B: unpaired *t*‐test: **p ≤ 0.01, ***p ≤ 0.001, ****p ≤ 0.0001.

Fibroblasts from patients with *HADHA* variants—FB822 (homozygous common LCHAD variant) and FB847 (compound heterozygous variants leading to complete TFP deficiency)—showed higher ROS levels than control fibroblasts (FB826), whereas FB861 cells with compound heterozygous *HADHB* variants had ROS levels comparable to control cells (Figure 5A). In FB822, elamipretide at 12.5 nM significantly reduced ROS, but this effect was not sustained at 25 nM. In FB847, ROS decreased at both concentrations. In contrast, FB861 cells showed no response to elamipretide. In βTFP‐deficient mice, liver mitochondria had significantly higher ROS levels compared to WT animals, which were reduced by elamipretide (Figure 5B).

### 3.6 CL Levels and Pattern Were Altered in Liver Mitochondrial Extracts From βTFP‐Deficient Mice and Did Not Change With Elamipretide Treatment

**FIGURE 6:**
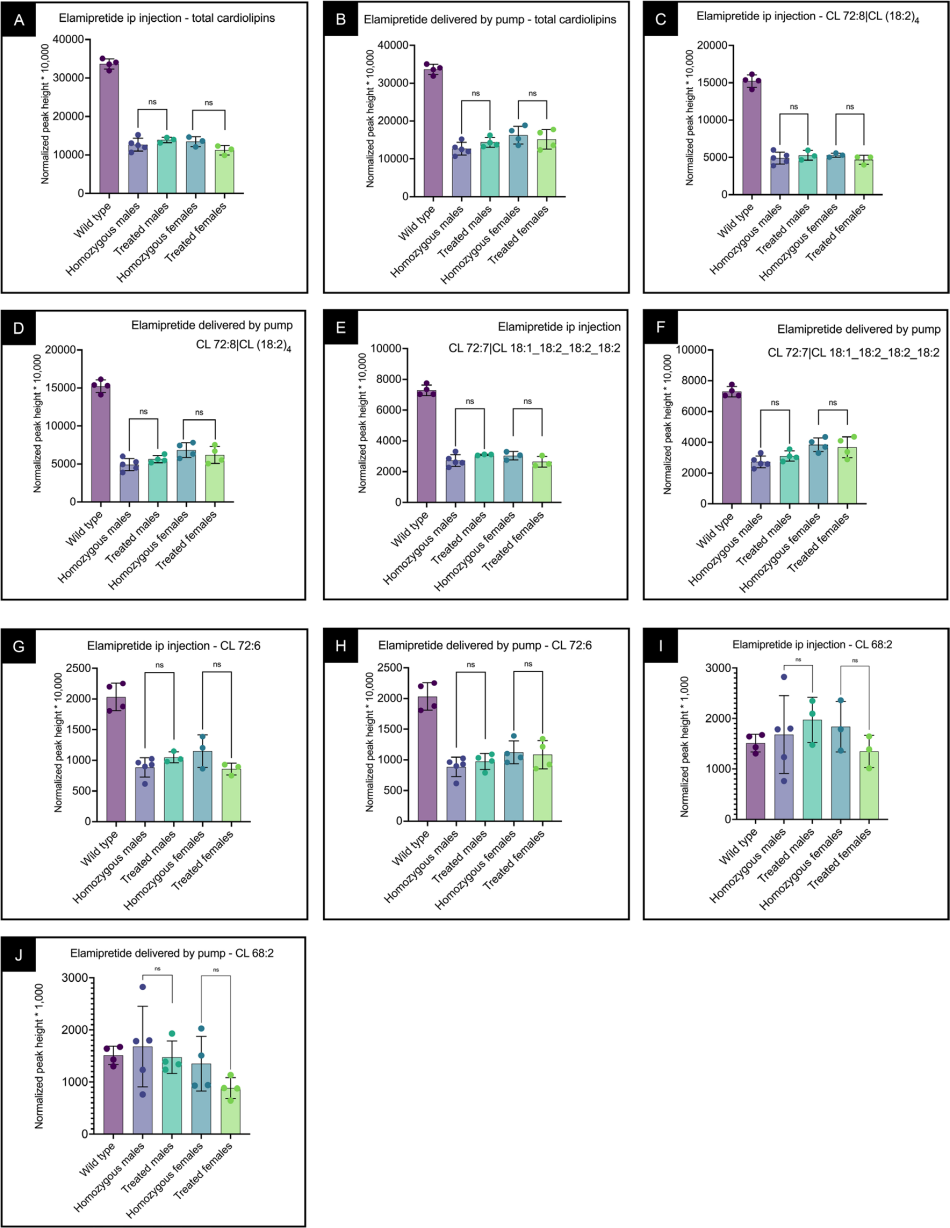
Cardiolipins in liver mitochondria extracts from male and female βTFP mutant mice treated with elamipretide delivered by IP injection or by osmotic minipumps. (A, B) Total cardiolipins in βTFP mutant mice treated with elamipretide by IP injection (A) or osmotic minipumps (B). All groups had significantly reduced levels compared to wild‐type (WT) mice. Elamipretide did not increase its levels when delivered by either route. (C, D) Tetralinoleoylcardiolipin (CL(72:8) | CL(18:2)_4_), the most abundant cardiolipin in mouse liver, was reduced by ~66% in βTFP males and ~57% in females. Elamipretide did not increase its levels when delivered by either route. (E–H) Other 72‐carbon polyunsaturated cardiolipins, including CL(72:7) (E, F) and CL(72:6) (G, H), were also unchanged by treatment. (I, J) Cardiolipins with shorter and more saturated acyl chains, such as CL(68:2), showed similar levels in WT and βTFP mice. Older (6 months) females treated by osmotic minipumps had reduced levels compared to WT and similarly treated males, but not to control females (J). In younger females (~3 months) treated by IP injection, CL(68:2) levels did not differ significantly from those of WT, treated males, or control females (I). Overall, elamipretide did not alter total cardiolipin levels or the distribution of individual cardiolipin species. Comparisons were performed using an unpaired *t*‐test. *p* values: ns *p* > 0.05, *p ≤ 0.05, **p ≤ 0.01, ***p ≤ 0.001, and ****p ≤ 0.0001.

We have previously reported reduced CL levels in mitochondria of βTFP‐deficient mice [23]. Consistently, total CLs in liver mitochondrial extracts were reduced in βTFP mutant mice (Figure 6A,B). Tetralinoleoylcardiolipin (CL(72:8) | CL(18:2)_4_), the most abundant CL in mouse liver, was reduced by 66% in βTFP‐deficient males and 57% in females. Elamipretide, delivered either by IP injection (Figure 6A) or osmotic minipump (Figure 6B), did not increase CLs, including CL(72:8) (Figure 6C,D), or other 72‐carbon polyunsaturated CLs such as CL(72:7), the second most abundant CL (Figure 6E–H). CLs with shorter, more saturated fatty acyl chains, such as CL(68:2) (19 possible acyl chain compositions), were similar between βTFP‐deficient and WT animals and unaffected by elamipretide treatment (Figure 6I,J) [43]. Females treated with elamipretide by osmotic minipump had lower CL(68:2) than WT and treated males, but not lower than untreated females (Figure 6J); these females were older (~6 months) than those treated by IP injection (~3 months).

**FIGURE 7:**
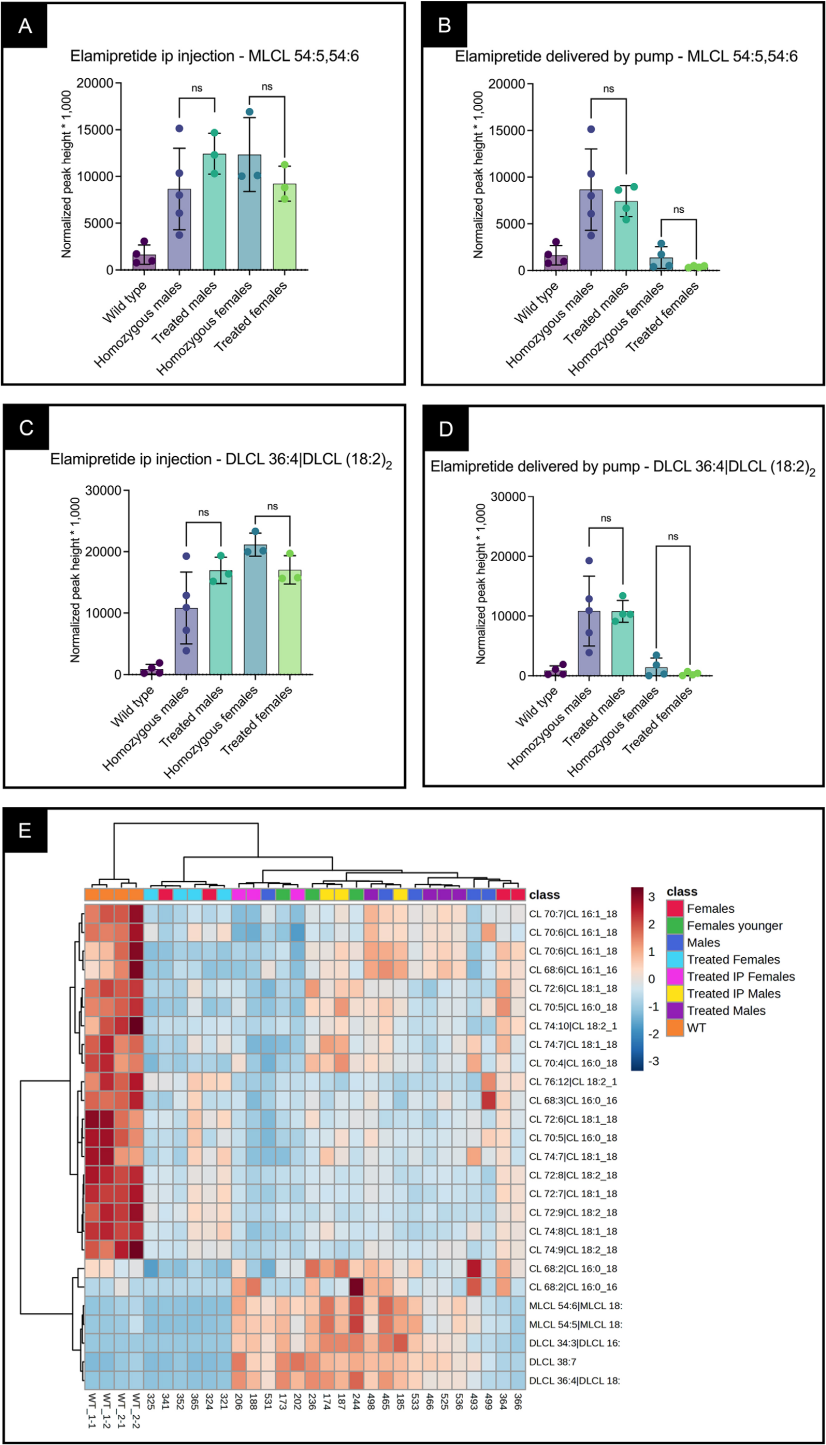
Monolysocardiolipins (MLCLs) and dilysocardiolipins (DLCLs) in liver mitochondria extracts from male and female βTFP mutant mice treated with elamipretide by IP injection or osmotic minipumps. (A, B) MLCLs were elevated in βTFP males (~6–7 months) and females (~3 months) compared to wild‐type (WT) mice, but not in older females (6 months). Elamipretide did not significantly change MLCL levels when delivered by IP injection (A) or by osmotic minipumps (B). (C, D) DLCLs showed a similar pattern, with increases in βTFP males and younger females, but not in older females. Elamipretide treatment again had no significant effect. (E) Two‐dimensional clustering of cardiolipins, MLCLs, and DLCLs distinguished WT profiles from all βTFP groups and also segregated samples by age and sex. Elamipretide did not substantially alter overall CL/MLCL/DLCL clustering patterns, consistent with the lack of effect on individual species. Comparisons were performed using an unpaired *t*‐test. *p* values: ns *p* > 0.05, *p ≤ 0.05, **p ≤ 0.01, ***p ≤ 0.001, and ****p ≤ 0.0001.

Monolysocardiolipins (MLCLs) and dilysocardiolipins (DLCLs) were elevated in βTFP‐deficient males (~6–7 months) and younger females (~3 months) compared to WT animals, consistent with impaired MLCLAT function, whereas older females (~6 months) showed levels similar to WT animals, as previously reported [23]. Elamipretide, delivered by either route, did not significantly change the levels of these phospholipids in any group (Figure 7A–D).

Two‐dimensional clustering of CLs, MLCLs, and DLCLs showed a distinct WT profile compared to all βTFP‐deficient groups. Age and sex also segregated samples—3 months females (IP and untreated) and 6–7 months males (IP, minipump, and untreated) versus 6 months females (minipump and untreated). Elamipretide treatment did not alter overall CL/MLCL/DLCL profiles in βTFP‐deficient mice (Figure 7E), consistent with the lack of effect on individual CL species shown in Figure 6.

### 3.7 Elamipretide Increased Mitochondrial Bioenergetics in TFP/LCHAD‐Deficient‐Patient Derived Fibroblasts

**FIGURE 8:**
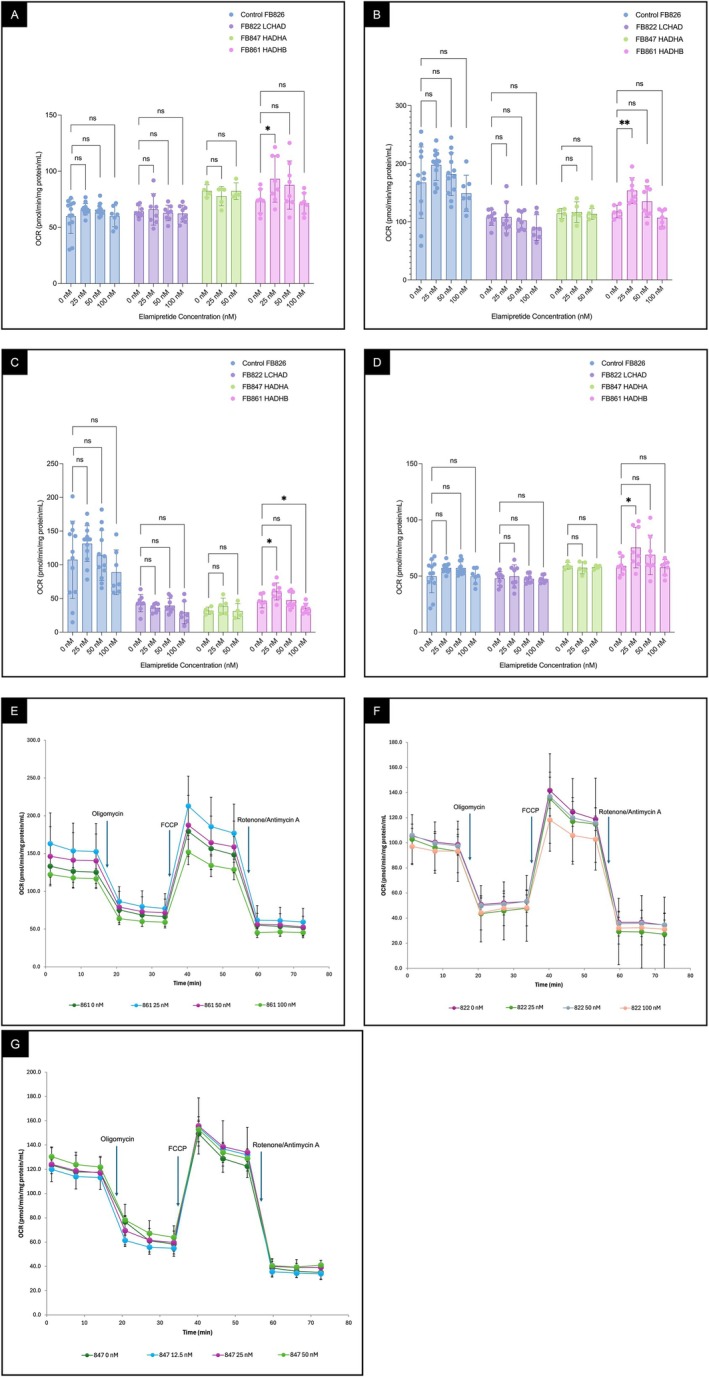
Mitochondrial bioenergetics parameters of human fibroblasts after elamipretide treatment (25–100 or 25–50 nM) evaluated using a Seahorse XFe96 Extracellular Flux Analyzer. Two assays were performed for control cells and one for TFP/LCHAD‐deficient cells, each with at least four biological replicates. FB861, fibroblasts from a compound heterozygous *HADHB* male patient; FB822, fibroblasts from a homozygous *HADHA* p.E510Q (isolated LCHAD deficiency) male patient; FB847, fibroblasts from a compound heterozygous *HADHA* (generalized TFP deficiency) male patient; FB826, fibroblasts from an unaffected female control. (A) Basal respiration. (B) Maximal respiration. (C) Spare respiratory capacity. (D) ATP‐linked respiration. (E–G) Time‐course graphs of oxygen consumption rate (OCR) for fibroblasts treated with different elamipretide concentrations, showing distinct phases after the injection of mitochondrial inhibitors: FB861 (E), FB822 (F), and FB847 (G). Comparisons were performed using an unpaired *t*‐test. *p* values: ns *p* > 0.05, *p ≤ 0.05, **p ≤ 0.01, ***p ≤ 0.001, and ****p ≤ 0.0001.

FB861 patient‐derived fibroblasts, carrying compound heterozygous *HADHB* variants, showed bioenergetic deficits with reduced maximal respiration (Figure 8B) and spare respiratory capacity (Figure 8C), while basal respiration (Figure 8A) and ATP‐linked respiration (Figure 8D) were unchanged compared to control cells (FB826), as assayed in a Seahorse bioanalyzer. All parameters improved after 72 h incubation with 25 nM elamipretide. FB822 fibroblasts, homozygous for the common *HADHA* p.E510Q variant, and FB847 fibroblasts, with compound heterozygous *HADHA* variants, exhibited similar bioenergetic deficits (Figure 8B,C), but none of these parameters improved with elamipretide treatment. In a second assay, fibroblasts from a male and a female homozygous for HADHA p.E510Q variant (FB822 and FB830, respectively) both showed decreases in all cellular bioenergetics parameters, more pronounced in the female cells, with no improvement after treatment (Figure S1A–D). Of note, in a third assay, FB822 fibroblasts again failed to show improvement with higher elamipretide doses and displayed some toxicity at doses ≥ 100 nM (Figure S2A–D). Time‐course graphs of OCR over time, showing distinct phases after the injection of mitochondrial inhibitors, are shown in Figure 8E–G.

## 4 Discussion

Long‐chain fatty acid oxidation disorders (LC‐FAODs) are clinically characterized by hypoglycemia, cardiomyopathy, and/or recurrent rhabdomyolysis. In addition, patients with TFP/LCHAD deficiency develop peripheral neuropathy and/or retinal pigmentary degeneration (RPD); however, the reasons for these clinical differences remain unknown. A paradox exists in the high incidence of RPD among patients with the common *HADHA* c.1528G>C (p.E510Q) variant, despite the retina being primarily a glycolytic organ [44]. Cardiomyopathy in LC‐FAODs is typically attributed to a combination of lipid droplet accumulation, impaired ATP production, and the buildup of toxic 3‐hydroxyacylcarnitines [45, 46, 47, 48, 49]. Peripheral neuropathy and RPD have similarly been attributed to the accumulation of these metabolites [44, 50, 51, 52]. However, these proposed pathogenetic mechanisms do not explain why some patients develop these symptoms while others do not, since elevated 3‐hydroxyacylcarnitine levels have been found in patients with TFP deficiency without RPD, whereas mildly affected mitochondrial bioenergetics have been observed in patients with cardiomyopathy [23]. Unfortunately, the current standard of care for LC‐FAODs, including avoidance of fasting, a low‐fat diet, and supplementation with medium‐chain triglycerides (odd‐ or even‐chain), has little effect on the development of neurological or ophthalmologic manifestations of TFP/LCHAD deficiency.

Recognition of a fourth enzymatic function in TFP involved in CL metabolism, associated with reduced CL levels, abnormal CL profiles, and CL peroxidation, offers an opportunity to explore its contribution to the unique clinical phenotype of TFP/LCHAD deficiency, and to develop novel therapeutic approaches [23]. Elamipretide is a mitochondria‐targeted tetrapeptide that selectively interacts with CL, protecting it from oxidative damage through combined electrostatic and hydrophobic interactions. This protection prevents oxidized CL from converting cytochrome *c* into a peroxidase, thereby maintaining its role as an electron carrier [14, 53, 54]. CL also interacts with and stabilizes the individual ETC complexes and their assembly into supercomplexes [12, 55, 56, 57, 58]. Given the role of TFP in CL metabolism and the abnormalities in CL quantity and pattern observed in TFP‐LCHAD deficiency, elamipretide is a logical therapeutic candidate [23].

The mouse model used here carries the p.M404K missense mutation in *Hadhb*. Structural mapping indicates that this substitution is not located in the β‐subunit interface regions of the α1‐β1 [H226‐F235 (human H225‐F234) and K392‐Y403 (human K391‐Y402)] or the α1‐β2 [H78‐S86 (human H77‐T85), E119‐G125 (human E118‐G124), K269‐G272 (human K268‐G271), and K273‐T274 (human K272‐T274)] interactions [5, 59, 60]. It is also outside the membrane‐binding helices H4 (S177‐D186, human S176‐D185) and H5 (L193‐F207, human M192‐F206). These features argue against direct disruption of α‐β contacts or membrane anchoring. Instead, p.M404K inserts a charged residue that likely reduces β‐subunit stability or folding efficiency, thereby impairing heterotetramer formation, FAO‐ETC interaction, and ultimately CL remodeling. Supporting this interpretation, we previously demonstrated reduced CL levels in liver mitochondria from this model [23]. The present findings further support evaluating CL‐stabilizing agents such as elamipretide, as both patient‐derived cells and the mouse model display CL abnormalities. It is plausible that elamipretide interacts with CL in the IMM to promote the stability of mitochondrial enzyme complexes and enhance bioenergetics function. This mechanism aligns with prior reports that elamipretide improves the activities of ETC complexes and supercomplexes, particularly enhancing complex I activity and FAO‐ETC bridging [13, 31].

Differences in some CL parameters, especially regarding MLCL elevation, in male and female patient‐derived TFP/LCHAD‐deficient cell lines and βTFP‐deficient mice [23], underscore the importance of considering sex as a biological variable in preclinical and translational studies of elamipretide. The mechanistic basis of these differences remains unclear, but sex‐specific variations in mitochondrial function have been increasingly reported in rodents and humans [61, 62, 63, 64, 65].

Although elamipretide improved mitochondrial bioenergetics in our βTFP‐deficient models, we did not detect changes in total CL content or in MLCL/DLCL abundance in liver mitochondria. At first glance, this may seem inconsistent with a mechanism based on “CL stabilization.” However, accumulating evidence indicates that elamipretide can enhance mitochondrial function without altering total CL levels [66, 67, 68]. Its actions likely include protection of CL from peroxidation, preservation of cytochrome *c* electron‐carrier function [14, 66], facilitation of ETC supercomplex assembly, and redistribution of CL within the IMM through PLSCR3‐dependent remodeling [69]. These effects can restore respiratory efficiency and reduce oxidative stress independently of overall CL quantity. The reduction in ROS observed in mouse liver mitochondria and in certain patient fibroblasts, although not a direct measure of lipid peroxidation, provides indirect support for the hypothesis that this mechanism could be partially responsible for the observed benefits. Importantly, quantification of total CL in bulk mitochondrial extracts may obscure subtle but biologically significant changes in CL acyl‐chain composition, oxidation status, or sub‐mitochondrial localization. Thus, the observed physiological benefits of elamipretide in TFP deficiency are consistent with its established modes of action, even in the absence of measurable increases in total CL content.

In fibroblasts, ROS levels were measured with MitoSOX Red, which detects mitochondrial superoxide in intact cells, whereas in isolated liver mitochondria, DCFH‐DA was used to measure general oxidant production. Although these complementary assays are not directly comparable, both consistently showed a reduction in oxidant signals after elamipretide treatment. Because only one *HADHB*‐deficient patient line (FB861) was available, the observed improvement in respiratory parameters should be confirmed in additional cell lines.

Due to the modest sample size in the treadmill experiments, our findings should be considered as preliminary, though they are supported by the positive results with skeletal muscle mitochondria. Further evaluation of elamipretide in this disease will require larger sample sizes and additional functional assessments, such as grip strength and wire‐hang tests. Investigation of the effect of elamipretide on the cardiomyopathy known to occur in βTFP‐deficient mice will require a longer‐term dedicated study.

In summary, elamipretide treatment of βTFP‐deficient mice and patient‐derived fibroblasts demonstrated improvement in cellular physiology and bioenergetics without consistently altering total CL content or MLCL/DLCL profiles. These findings support a mechanism whereby elamipretide enhances mitochondrial function through effects that extend beyond the simple restoration of CL levels. Collectively, our results identify elamipretide as a promising therapeutic candidate for TFP/LCHAD deficiency.

## Author Contributions

Conceptualization and design: E.V.N., Y.W., and J.V. Methodology: E.V.N., S.B., Y.W., and J.V. Animal Experiments: E.V.N., M.W., L.F., S.B., and X.J.Z. Fibroblast studies: E.V.N., A.J.S., and A.K. Biochemical assays and lipidomics: E.V.N. and Y.W. Formal analysis: E.V.N., Y.W., and J.V. Writing – original draft: E.V.N. and J.V. Writing – review and editing: All authors. Funding acquisition: E.V.N. and J.V. Resources: E.V.N., S.B., Y.W., and J.V. Supervision: J.V.

## Funding

This work was supported by the Children's Neuroscience Institute Interdisciplinary Neuroscience Award research grant and the UPMC Children's Hospital of Pittsburgh Foundation.

## Ethics Statement

Human fibroblast cell lines from patients confirmed to have TFP/LCHAD deficiency have been previously obtained for other clinical testing, so no procedures specific to this study were performed. All animal studies were conducted in accordance with research protocols approved by the University of Pittsburgh IACUC (Protocol IS00020939).

## Consent

Consent to provide the samples was obtained in accordance with the referring physicians' institutional review boards. For patients from the Children's Hospital of Pittsburgh, informed consent was obtained for testing and storage of a fibroblast or tissue sample as approved by our IRB under Protocol CR19030195‐010.

## Conflicts of Interest

The authors declare no conflicts of interest.

## Supporting information


**Table S1:** Clinical characteristics of patients with TFP/LCHAD deficiency included in the study.
**Table S2:** TFP/LCHAD‐deficient fibroblast cell lines and their respective genotypes.
**Table S3:** Control dermal fibroblast cell lines obtained from apparently healthy individuals used in this study.
**Figure S1:** Mitochondrial bioenergetics parameters of human fibroblasts after elamipretide treatment in the concentration ranges of 25–100 nM, evaluated by a Seahorse XFe96 Extracellular Flux Analyzer. There were at least three biological replicates. FB822, fibroblasts from a homozygous HADHA isolated LCHAD p.E510Q deficient male patient; FB830, fibroblasts from a female patient with the same genotype as FB822; FB826, fibroblasts from an unaffected female individual. (A) Basal respiration. (B) Maximal respiration. (C) Spare respiratory capacity. (D) ATP‐linked respiration. Comparisons were performed using a two‐way ANOVA followed by Šídák's multiple comparisons test. *p* values: ns *p* > 0.05, *p ≤ 0.05, **p ≤ 0.01, ***p ≤ 0.001, and ****p ≤ 0.0001.
**Figure S2:** Mitochondrial bioenergetics parameters of human fibroblasts after elamipretide treatment in the concentration ranges of 10 nM to 1 μM, evaluated by a Seahorse XFe96 Extracellular Flux Analyzer. There were at least seven biological replicates. FB822, fibroblasts from a homozygous HADHA isolated LCHAD p.E510Q deficient male patient; FB902, fibroblasts from an unaffected male individual. (A) Basal respiration. (B) Maximal respiration. (C) Spare respiratory capacity. (D) ATP‐linked respiration. Comparisons were performed using a two‐way ANOVA followed by Šídák's multiple comparisons test. *p* values: ns *p* > 0.05, *p ≤ 0.05, **p ≤ 0.01, ***p ≤ 0.001, and ****p ≤ 0.0001.
**Figure S3:** Full unprocessed image of in‐gel complex V activity in liver mitochondria of βTFP mutant mice. NP, non‐pertinent; Treat, treated; Untr, untreated; WT, wild type.

## Data Availability

The data that support the findings of this study are available from the corresponding author upon reasonable request.
