# Supplementary material for: Elamipretide Improves Mitochondrial Function in Mitochondrial Trifunctional Protein‐Deficient Mice and Human Fibroblasts
Source: J Inherit Metab Dis. 2026 Jan 7;49(1):e70132. doi: 10.1002/jimd.70132 (PMC12779498; doi:10.1002/jimd.70132)
Supplement: Supplementary file 1 — Table S1: Clinical characteristics of patients with TFP/LCHAD deficiency included in the study. Table S2: TFP/LCHAD‐deficient fibroblast cell lines and their respective genotypes. Table S3: Control dermal fibroblast cell lines obtained from apparently healthy individuals used in this study. Figure S1: Mitochondrial bioenergetics parameters of human fibroblasts after elamipretide treatment in the concentration ranges of 25–100 nM, evaluated by a Seahorse XFe96 Extracellular Flux Analyzer. There were at least three biological replicates. FB822, fibroblasts from a homozygous HADHA isolated LCHAD p.E510Q deficient male patient; FB830, fibroblasts from a female patient with the same genotype as FB822; FB826, fibroblasts from an unaffected female individual. (A) Basal respiration. (B) Maximal respiration. (C) Spare respiratory capacity. (D) ATP‐linked respiration. Comparisons were performed using a two‐way ANOVA followed by Šídák's multiple comparisons test. p values: ns p > 0.05, *p ≤ 0.05, **p ≤ 0.01, ***p ≤ 0.001, and ****p ≤ 0.0001. Figure S2: Mitochondrial bioenergetics parameters of human fibroblasts after elamipretide treatment in the concentration ranges of 10 nM to 1 μM, evaluated by a Seahorse XFe96 Extracellular Flux Analyzer. There were at least seven biological replicates. FB822, fibroblasts from a homozygous HADHA isolated LCHAD p.E510Q deficient male patient; FB902, fibroblasts from an unaffected male individual. (A) Basal respiration. (B) Maximal respiration. (C) Spare respiratory capacity. (D) ATP‐linked respiration. Comparisons were performed using a two‐way ANOVA followed by Šídák's multiple comparisons test. p values: ns p > 0.05, *p ≤ 0.05, **p ≤ 0.01, ***p ≤ 0.001, and ****p ≤ 0.0001. Figure S3: Full unprocessed image of in‐gel complex V activity in liver mitochondria of βTFP mutant mice. NP, non‐pertinent; Treat, treated; Untr, untreated; WT, wild type. [file JIMD-49-0-s001.docx]

**Table S1.** Clinical characteristics of patients with TFP/LCHAD deficiency included in the study

| Patient | Cell line | Sex | Current state | TFP/LCHAD Deficiency | Hypoglycemia | Hypotonia/Motor Delay | Muscle weakness | Rhabdomyolysis | Cardiomyopathy | Peripheral neuropathy | Retinopathy | Maternal HELLP syndrome |
| --- | --- | --- | --- | --- | --- | --- | --- | --- | --- | --- | --- | --- |
| 1 | FB822 | M | 14y | LCHAD | N | N | N | Y | Y (reversed by treatment) | N | N | Y |
| 2 | FB830/ Coriell Institute GM20266 | F | ? | LCHAD | ? | ? | ? | ? | ? | ? | ? | ? |
| 3 | FB847 | M | 9y | TFP (*HADHA*) | N | N | Y | Y | N | N | N | Y |
| 4 | FB861 | M | 22y, wheelchair-bound | TFP (*HADHB*) | Y | Y | Y | Y | N | Y | N | N |

Legend: AFOs, Ankle-Foot Orthoses; HELLP syndrome, hemolysis, elevated liver enzymes, and low platelets syndrome

**Table S2.** TFP/LCHAD-deficient fibroblast cell lines and their respective genotypes

| Patient | Sex | TFP/LCHAD Deficiency | Cell line | Gene Affected | Allele 1 | Amino acid change | Reference sequence | Allele 2 | Amino acid change | Reference sequence |
| --- | --- | --- | --- | --- | --- | --- | --- | --- | --- | --- |
| 1 | M | LCHAD | FB822 | *HADHA* | c.1528G>C | p.E510Q | NM_000182.5 | c.1528G>C | p.E510Q | NM_000182.5 |
| 2 | F | LCHAD | FB830 | *HADHA* | c.1528G>C | p.E510Q | NM_000182.5 | c.1528G>C | p.E510Q | NM_000182.5 |
| 3 | M | TFP | FB847 | *HADHA* | c.2146+1G>A | — | NG_007121.1 | c.403A>G | p.K135E | NM_000182.5 |
| 4 | M | TFP | FB861 | *HADHB* | c.693delC | p.A232Lfs*20 | NM_000183.3 | c.881C>G | p.P294R | NM_000183.3 |

**Table S3.** Control dermal fibroblast cell lines obtained from apparently healthy individuals used in this study

| Cell line | Sex | Age (at sampling) | Origin | Skin source | Race/Ethnicity | Karyotype |
| --- | --- | --- | --- | --- | --- | --- |
| FB826 | F | 40 yr. | ATCC PCS-201-012 | Abdominal | White | Unspecified |
| FB902 | M | 22 yr. | Coriell GM23976 | Unspecified | White | Unspecified |

*
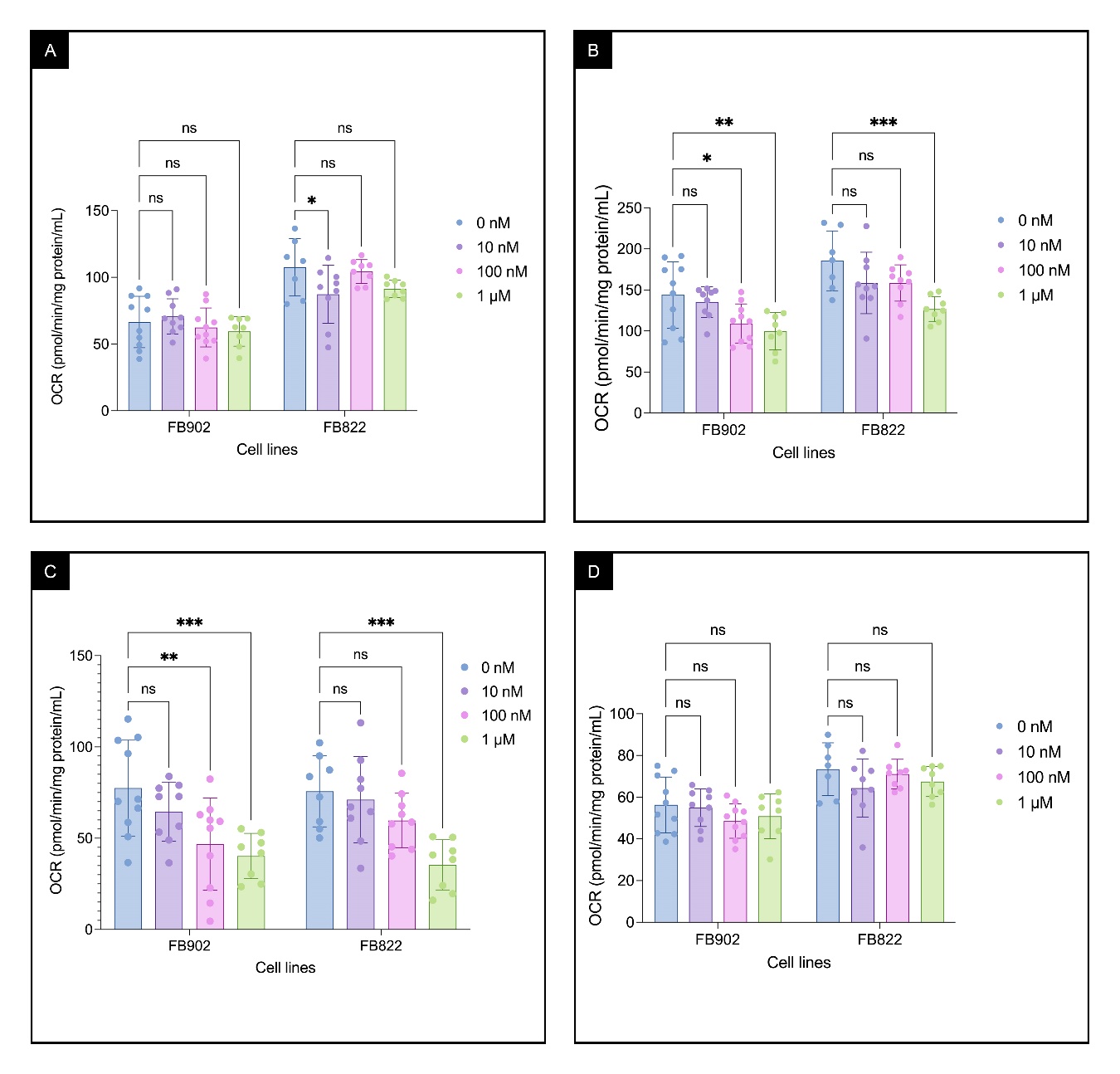
*

*Figure S1. Mitochondrial bioenergetics parameters of human fibroblasts after elamipretide treatment in the concentration ranges of 25-100 nM, evaluated by a Seahorse XFe96 Extracellular Flux Analyzer. There were at least 3 biological replicates. FB822, fibroblasts from a homozygous HADHA isolated LCHAD p.E510Q deficient male patient; FB830, fibroblasts from a female patient with the same genotype as FB822; FB826, fibroblasts from an unaffected female individual. (A) Basal respiration. (B) Maximal respiration. (C) Spare respiratory capacity. (D) ATP-linked respiration. Comparisons were done by two-way ANOVA followed by Šídák's multiple comparisons test. P values: ns P>0.05, ∗P≤ 0.05, ∗∗P≤0.01, ∗∗∗P≤0.001, ∗∗∗∗P≤0.0001.*

*Figure S2. Mitochondrial bioenergetics parameters of human fibroblasts after elamipretide treatment in the concentration ranges of 10 nM to 1 μM, evaluated by a Seahorse XFe96 Extracellular Flux Analyzer. There were at least 7 biological replicates. FB822, fibroblasts from a homozygous HADHA isolated LCHAD p.E510Q deficient male patient; FB902, fibroblasts from an unaffected male individual. (A) Basal respiration. (B) Maximal respiration. (C) Spare respiratory capacity. (D) ATP-linked respiration. Comparisons were done by two-way ANOVA followed by Šídák's multiple comparisons test. P values: ns P>0.05, ∗P≤ 0.05, ∗∗P≤0.01, ∗∗∗P≤0.001, ∗∗∗∗P≤0.0001.*


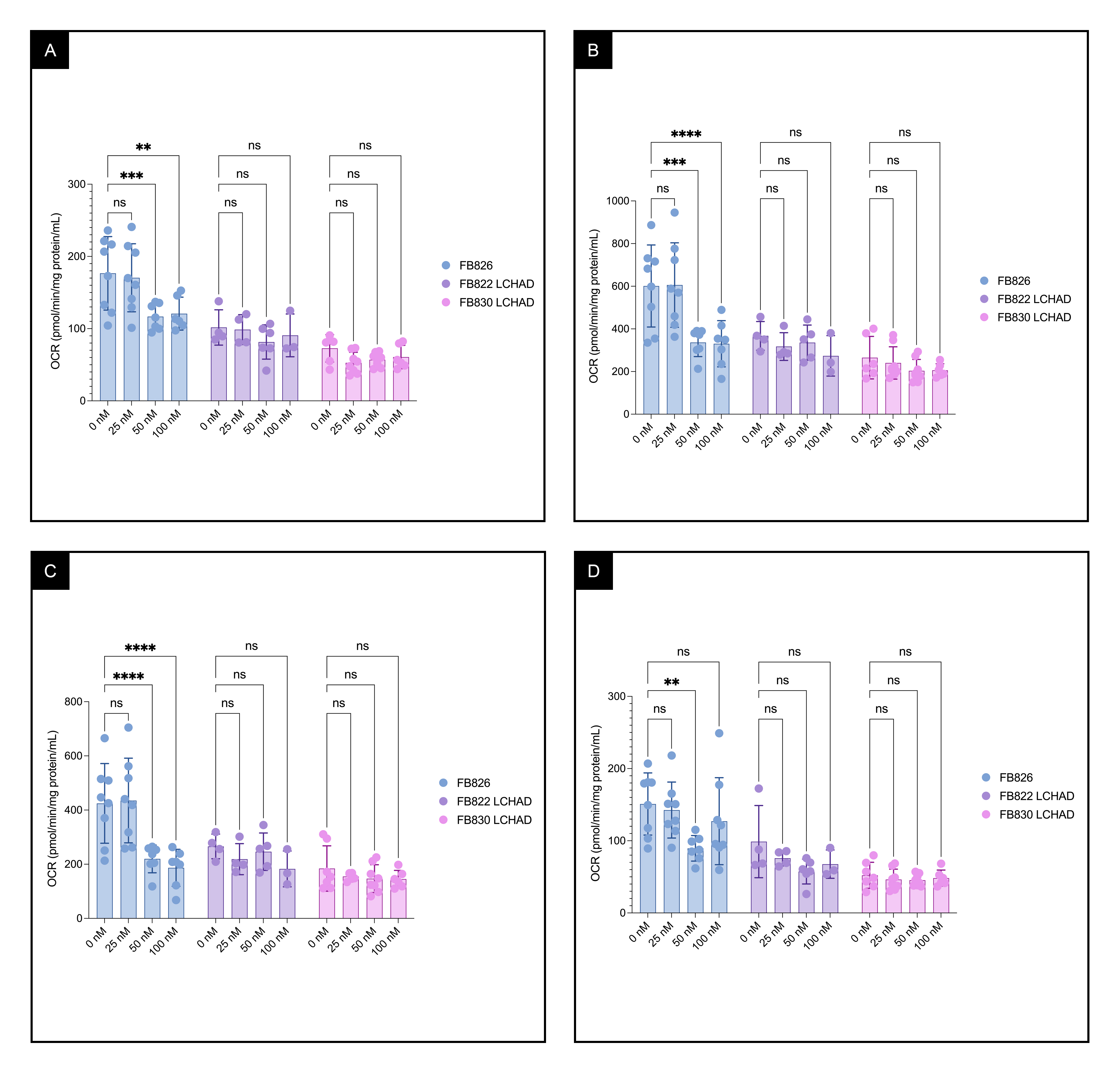


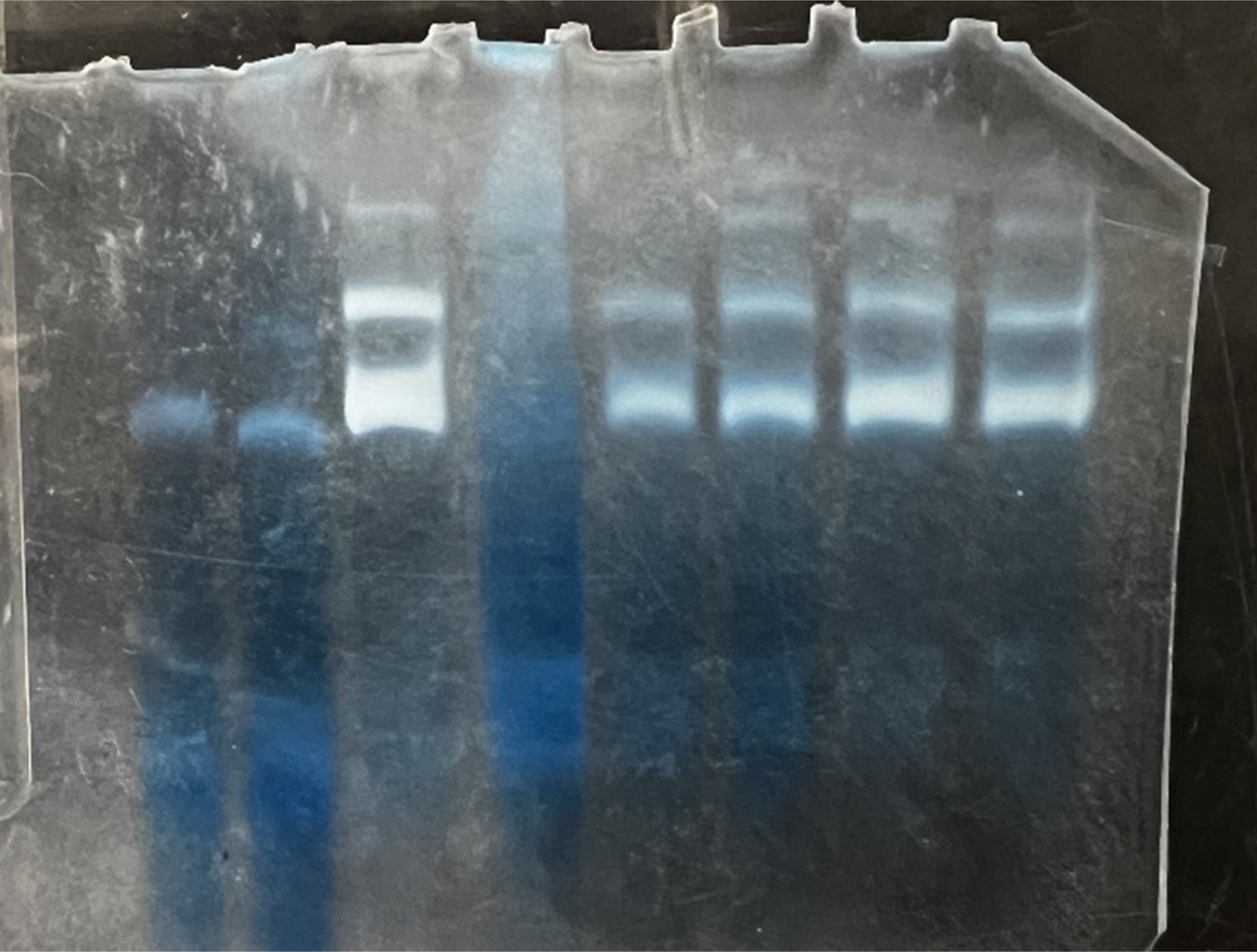


**NP NP WT NP Untr Untr Treat Treat**

*Figure S3. Full unprocessed image of in-gel complex V activity in liver mitochondria of βTFP mutant mice. NP, non-pertinent; WT, wild type; Untr, untreated; Treat, treated.*
